# Delayed Toxicity Associated with Soluble Anthrax Toxin Receptor Decoy-Ig Fusion Protein Treatment

**DOI:** 10.1371/journal.pone.0034611

**Published:** 2012-04-12

**Authors:** Diane Thomas, John Naughton, Christopher Cote, Susan Welkos, Marianne Manchester, John A. T. Young

**Affiliations:** 1 Skaggs School of Pharmacy and Pharmaceutical Sciences, University of California San Diego, La Jolla, California, United States of America; 2 Nomis Center for Immunobiology and Microbial Pathogenesis, The Salk Institute for Biological Studies, La Jolla, California, United States of America; 3 Bacteriology Division, U.S. Army Medical Research Institute of Infectious Diseases, Fort Detrick, Frederick, Maryland, United States of America; Wadsworth Center, New York State Dept. Health, United States of America

## Abstract

Soluble receptor decoy inhibitors, including receptor-immunogloubulin (Ig) fusion proteins, have shown promise as candidate anthrax toxin therapeutics. These agents act by binding to the receptor-interaction site on the protective antigen (PA) toxin subunit, thereby blocking toxin binding to cell surface receptors. Here we have made the surprising observation that co-administration of receptor decoy-Ig fusion proteins significantly delayed, but did not protect, rats challenged with anthrax lethal toxin. The delayed toxicity was associated with the *in vivo* assembly of a long-lived complex comprised of anthrax lethal toxin and the receptor decoy-Ig inhibitor. Intoxication in this system presumably results from the slow dissociation of the toxin complex from the inhibitor following their prolonged circulation. We conclude that while receptor decoy-Ig proteins represent promising candidates for the early treatment of *B. anthracis* infection, they may not be suitable for therapeutic use at later stages when fatal levels of toxin have already accumulated in the bloodstream.

## Introduction

Anthrax toxin is the major virulence factor of *B. anthracis*, the causative agent of anthrax. There are two forms of anthrax toxin, each of which contains the protective antigen (PA) toxin subunit. Edema toxin (EdTx) pairs PA with edema factor (EF), a calcium and calmodulin-dependent adenylate cyclase; and lethal toxin (LeTx) is comprised of PA and lethal factor (LF), a zinc-dependent metalloprotease that cleaves and inactivates MAP kinase kinase signaling pathways [1], [2], [3].

The first step of intoxication involves binding of an 83 kD form of PA (PA_83_) to cell surface receptors. Two different cellular receptors for PA_83_ have been identified, designated as ANTXR1 and ANTXR2 [4], [5]. PA_83_ binds to an extracellular domain of each receptor that is related in structure to the integrin-like von Willebrand Factor type A (VWA) domain [4], [5]. Following receptor-binding, PA_83_ is cleaved to a 63 kD form by a cell surface furin-like protease, and the resultant PA_63_ fragment spontaneously assembles into either heptameric or octameric prepore complexes [6], [7], [8], [9]. Alternatively, these oligomeric PA complexes may assemble prior to receptor binding following PA_83_ to PA_63_ cleavage by a serum protease within the bloodstream of infected animals [10], [11]. The toxin complexes are taken up into cells by receptor-mediated endocytosis [12], [13], [14], [15]. Entry into an acidic endosomal compartment stimulates PA_63_ prepore-to-pore conversion and LF and EF translocation into the cytosol leading to toxicity [16].

There is a great deal of interest in developing anthrax antitoxins (reviewed in [17]). The rationale for developing these inhibitors is that they may complement existing vaccine and antibiotic-based therapies, and may be especially useful to treat disease caused by either vaccine-, or antibiotic-resistant bacterial strains. Several different anthrax anti-toxins are being developed including monoclonal antibodies, small molecule inhibitors, receptor decoys, substrate analogs, and dominant-negative toxin subunits.

We first demonstrated the utility of a soluble receptor decoy as a candidate anthrax therapeutic [18]. That inhibitor, based upon the soluble VWA domain of ANTXR2, had several desirable features including a high binding affinity for PA (Kd = 170 pM) [19]. The receptor decoy inhibitor also efficiently neutralized both wild-type PA, as well as altered forms of PA that were engineered to be resistant to therapeutic monoclonal antibodies [20], [21].

Several other groups have generated receptor decoy inhibitors by fusing the ANTXR2 VWA domain to the Fc portions of either human IgG1 or IgG2 [21], [22]. These reagents have the additional benefit of having an increased circulation half-life *in vivo*, thereby increasing their antitoxin potencies. The receptor decoy-IgG2 protein protected rats against short-term intoxication by anthrax lethal toxin, and also protected mice against killing following intratracheal administration of attenuated *B. anthracis* Sterne spores [22]. The receptor decoy-IgG1 protein protected rabbits against killing following an inhalational challenge with fully virulent *B. anthracis* Ames spores [21].

In this study we also generated and tested several different receptor decoy-Ig fusion proteins *in vitro* and *in vivo*. In contrast to previous reports, we found that these inhibitors delayed killing, but did not protect, rats that were challenged with a lethal dose of anthrax lethal toxin. The mechanism of delayed toxicity was studied by following the fate of the toxin subunits following co-administration with anthrax lethal toxin.

## Results

Our previous studies showed that a soluble, bacterially-produced form of the ANTXR2 VWA domain (termed receptor decoy inhibitor, or RDI) protected against LeTx challenge in rats, but only when it was administered concurrently or within 5 minutes of dosing with toxin [18]. That RDI protein was comprised of ANTXR2 amino acid residues 1–232 fused to a MycHis epitope tag [18].

**Figure 1:**
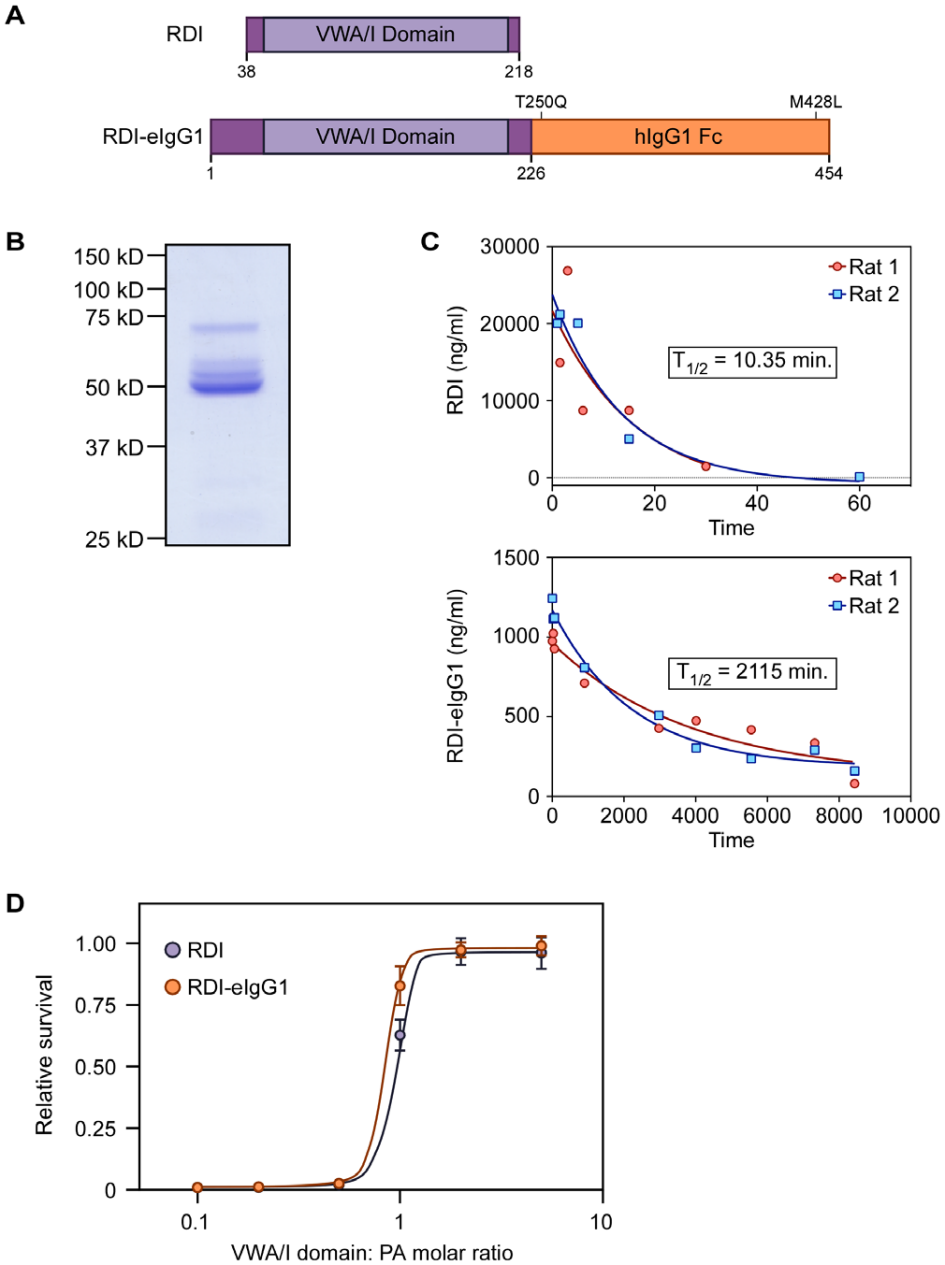
Construction, expression and half-life of RDI and RDI-eIgG1. A. Schematic of RDI and RDI-eIgG1 constructs. VWA/I: vonWillebrand Factor A/Integrin-like I domain. Mutations T250Q and M428L in the Fc portion confer extended circulation time. B. SimplyBlue (Invitrogen) stained gel of RDI-eIgG1 separated on a SDS-containing10% polyacrylamide gel. Molecular weight markers are indicated. C. Detection of RDI (top panel) or RDI-eIgG1 (bottom panel) after intravenous injection in 2 rats by ELISA analysis. Curve-fitting and half-life (t_1/2_) was calculated using Prism (Graph Pad, Inc.) D. Inhibition of LeTx activity in RAW264.7 cells by RDI or RDI-eIgG1.

In an effort to improve the potency of the receptor decoy we set out to generate a long-lived fusion protein comprised of the ANTXR2 VWA domain fused to the Fc portion of an IgG1 antibody, since that is a commonly used strategy to increase serum half-life of proteins. The Fc portion used contained two amino acid mutations (T250Q and M482L) that further extend the serum half-lives of IgG1 fusion proteins by approximately 2-fold [23]. The recombinant receptor decoy-Fc gene, designated as RDI-eIgG1 encoded amino acid residues 1 to 226 of ANTXR2 fused to the Fc portion of the altered IgG1 fragment in plasmid pFUSE-hIgG1e1-Fc1 (Invivogen) (Figure 1A). This DNA construct was transiently transfected into human 293 cells, and the approximately 50 kD RDI-eIgG1 fusion protein was purified from the extracellular supernatants by protein A-chromatography (Figure 1B).

The plasma half-life of the RDI-eIgG1 protein was compared in rats with that of the bacterially produced RDI [19]. Rats were injected intravenously with these proteins. Animals were bled at varying times post-dosing as shown in Figure 1C. Plasma concentrations were measured using a quantitative ELISA capture assay for the ANTXR2 VWA domain. For the RDI protein, the protein demonstrated first-order elimination and the t_1/2_ was shown to be 10.4 min (Figure 1C); calculated volume of distribution 48.6 ml; clearance 3259 ml/min, and area under the concentration-time curve (AUC) 282.5 µg-min/ml. In contrast, the RDI-eIgG1 protein showed a much longer t_1/2_ of 2115 min (∼35 hrs); volume of distribution 39.3 ml; clearance 12.9 ml/min, and AUC of 3.9×10^6^ µg-min/ml. The results of t_1/2_ and clearance measurements confirm that the RDI-eIgG1 protein circulated in plasma for much longer than the RDI protein, and the similarities in volume of distribution suggest that RDI-eIgG1 likely did not distribute to other tissues or sites in comparison to RDI.

**Figure 2:**
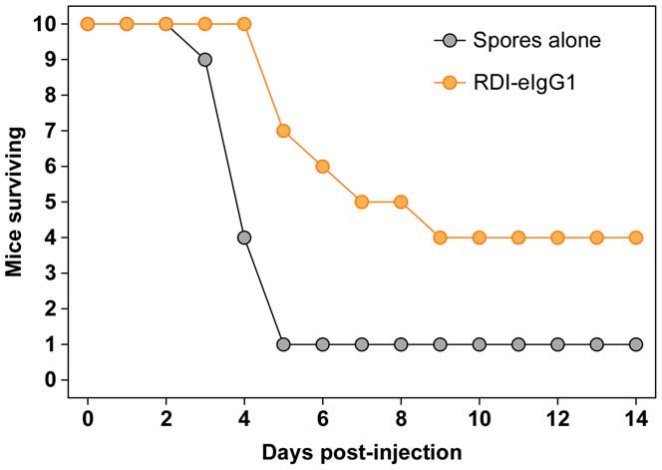
Survival of mice following spore challenge. Female A/J mice (8–10 weeks) were given approximately 2.9×10^4^ Sterne strain spores delivered in 100 µl of water IP. The CMG2-IgG was delivered IP in approximately 100 µg doses at 1 h, 28 h, and 52 h post infection. A final 50 µg dose of CMG2-IgG was administered at 96 h post-infection. Statistically significant P values were achieved for mean time to death or euthanasia by T-test (P = 0.03), and log-rank Mantel-Cox survival curve (P = 0.009).

The RDI-eIgG1 protein was compared to RDI in an *in vitro* intoxication assay to establish its inhibitory activity as a receptor decoy. In this approach, inhibitors were mixed at varying molar ratios of (VWA/I∶PA) with a fixed amount of LF and added to RAW264.7 cells. Intoxication was measured using an assay that monitors cellular ATP levels as a measure of cell viability. Both inhibitors showed similar inhibition profiles (Figure 1D). Consistent with previous published reports [21], [22], the RDI-eIgG1 protein showed some efficacy in protecting A/J mice from death following intraperitoneal injection with a lethal dose of *B. anthracis* Sterne spores. (Figure 2).

**Figure 3:**
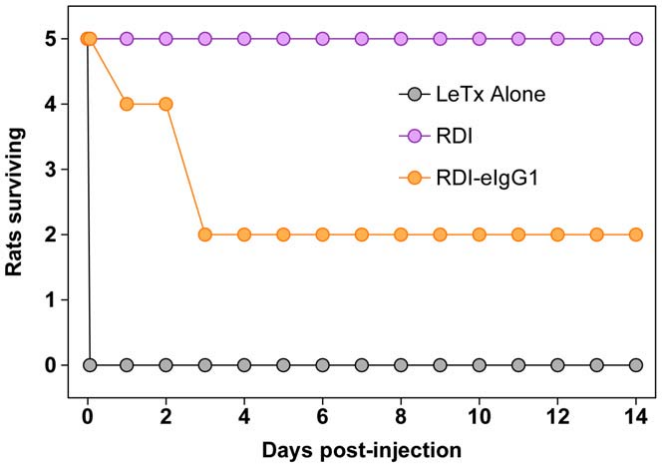
RDI-eIgG1 protects rats from LeTx challenge in the short term but shows delayed toxicity. Male 200 gram HSD rats (5/group) were co-injected i.v. with LeTx (40 µg PA+12 µg LF) and RDI (19 µg) or RDI-eIgG1 (49 µg). Rats were monitored and time of death recorded.

**Figure 4:**
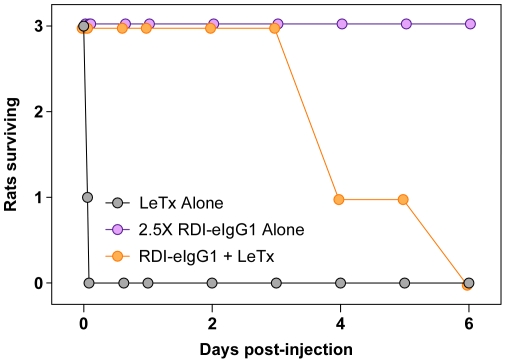
RDI-eIgG1 alone is not toxic in rats. Male 200 gram HSD rats (Harlan Laboratories, Indianapolis, IN) (3/group) were co-injected i.v. with LeTx (40 µg PA+12 µg LF) alone or in combination with 49 ug RDI-eIgG1; or were dosed i.v. with 123 µg RDI-eIgG1 alone. Rats were monitored and time of death recorded.

The RDI-eIgG1 fusion protein was also tested for its efficacy in protecting rats against challenge with a lethal dose of LeTx. Rats were injected i.v. with LeTx (40 µg of PA and 12 µg of LF) in combination with PBS, RDI, or RDI-eIgG1. Animals that had received LeTx alone died between 60 and 90 minutes after toxin challenge, while, as expected, all animals that received a 2∶1 molar ratio (RDI∶PA) and LF all survived (Fig. 3). Surprisingly, when the RDI-eIgG1 protein was co-injected with LeTx (at a similar 2∶1 ratio of ANTXR2 VWA-I domain: PA), the animals initially survived, but began to die approximately 20 hours after dosing (Fig. 3). By 3 days post-challenge, 50% of the animals in the group had died. This result was reproducible in six independent experiments (Table 1). Administration of a 2.5-fold higher dose of RDI-eIgG1 either alone, or in combination with PA, did not result in any morbidity, toxicity or pathology (Fig. 4), indicating that the complete LeTx (PA+LF) was required to produce the observed delayed toxicity. RDI-eIgG1 was also not found to be immunogenic in rats (data not shown). Together these results demonstrated that RDI-eIgG1, like RDI, provided initial protection when it was co-administered with a lethal dose of LeTx to animals. However, in contrast to the effective long-term protection seen with the short-lived RDI protein following co-administration with a fatal dose of toxin, the longer-lived RDI-eIgG1 protein was much less effective at providing long-term protection under this condition. These data indicated that the delayed time to death seen with the RDI-eIgG1 protein might be associated with its prolonged circulation half-life relative to RDI.

**Table 1:** Summary of studies measuring RDI-eIgG1 protection from LeTx challenge in rats.

Study #	Inhibitor	Survivors/Total	Average Time to Death (hr.)	Comparison of survival curve (log-rank Mantel-Cox)	Comparison of TTD unpaired t-test
1	RDI-eIgG1	2/5	56	P = 0.0003	P = 0.0033
	None	0/5	1		
2	RDI-eIgG1	0/3	112	P = 0.0246	P = 0.0023
	None	0/3	1.5		
3	RDI-eIgG1	0/5	52.8	P = 0.0027	P = 0.0364
	None	0/4	1.2		
4	RDI-eIgG1	1/5	90	P = 0.0049	P = 0.0001
	None	0/5	1.2		
5	RDI-eIgG1	0/5	77.6	P = 0.0019	P = 0.0015
	None	0/5	1.1		

Male 200 gram HSD were co-injected i.v. with LeTx (40 µg PA+12 µg LF) alone or in combination with RDI-eIgG1. Number of surviving animals in each study and the average time to death (in hours) is reported. Statistical analyses of survival curves (log-rank Mantel-Cox) and time-to-death (Student T-test) were performed using Prism (Graph Pad, Inc.).

Since PA-ANTXR2 complexes are extremely stable, with a lifetime interaction of approximately 30 hours *in vitro* [19], we reasoned that the delayed toxicity might be due to the slow release of oligomeric PA_63_/LF complexes that had assembled on the long-lived RDI-eIgG1 protein *in vivo*. To address this possibility, we asked whether LF and PA could be co-purified with RDI-eIgG1 at a late time point following co-administration. Rats were injected through jugular vein cannulas with 40 µg PA_83_, 12 µg LF, and 122 µg of RDI-eIgG1 (representing a 5∶1 molar ratio of ANTXR2 VWA/I domain∶PA). Blood was collected 15 hours after injection, serum was prepared, and immediately subjected to Protein G-sepharose precipitation (Roche Diagnostics, Mannheim, Germany) to purify RDI-eIgG1 and any associated proteins from the rat serum. Immunoprecipitated samples were subjected to SDS-PAGE and to immunoblotting using either a goat-anti-PA antibody (to detect PA_83_ or PA_63_) or a goat anti-LF antibody.

**Figure 5:**
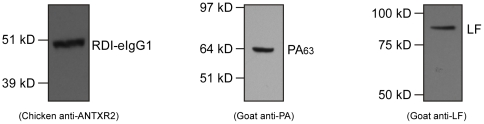
Association of PA_63_ and LF with RDI-eIgG1 during circulation *in vivo*. Rats were injected through jugular vein cannulas with a total volume of 500 µl containing 40 µg PA, 12 µg LF, 122 µg of RDI-eIgG1 in PBS vehicle per rat. This corresponds to a 5∶1 molar ratio of the VWA/I domain∶PA. Serum was prepared from blood collected 15 hours after injection and protein G sepharose was used to precipitate RDI-eIgG1 and associated proteins from the rat serum. Samples were separated on a SDS-containing 4–12% polyacrylamide gel and analyzed by immunoblotting for the presence of RDI-eIgG1, PA, or LF.

Both LF and the 63 kD form of PA were co-purified with RDI-eIgG1 (Figure 5). These studies indicated that a PA_63_/LF complex was associated with the RDI-eIgG1 protein in the rat circulation at 15 hours post-injection. PA_63_ is known to be generated *in vivo* from PA_83_ by serum protease cleavage [10], [11]. We conclude that the PA_63_ present in these complexes is in an oligomeric form, because LF is known to bind to oligomeric, but not monomeric, PA [24]. These findings are consistent with a model in which oligomeric LeTx complexes assemble on circulating RDI-eIgG1, and that the slow release of these assembled toxin complexes may be responsible for the delayed toxicity seen following co-administration of LeTx and RDI-eIgG1.

To determine whether this delayed toxicity was specific to the extremely long-lived RDI-eIgG1 protein, we generated other receptor decoy-Ig proteins containing the Fc portions of either wild-type IgG2 or IgG1. Vuyisich *et al.* previously showed that a receptor decoy-IgG2 fusion protein protected rats for a short period of time against LeTx challenge, although it was not reported whether longer time points after intoxication were studied [22]. That receptor decoy-IgG2 protein also protected mice against killing following intratracheal administration of attenuated *B. anthracis* Sterne spores [22]. A separate study by Wycoff *et al.* showed that a receptor decoy-IgG1 protein can protect rabbits against killing following an inhalational challenge with fully virulent *B. anthracis* Ames spores [21].

**Figure 6:**
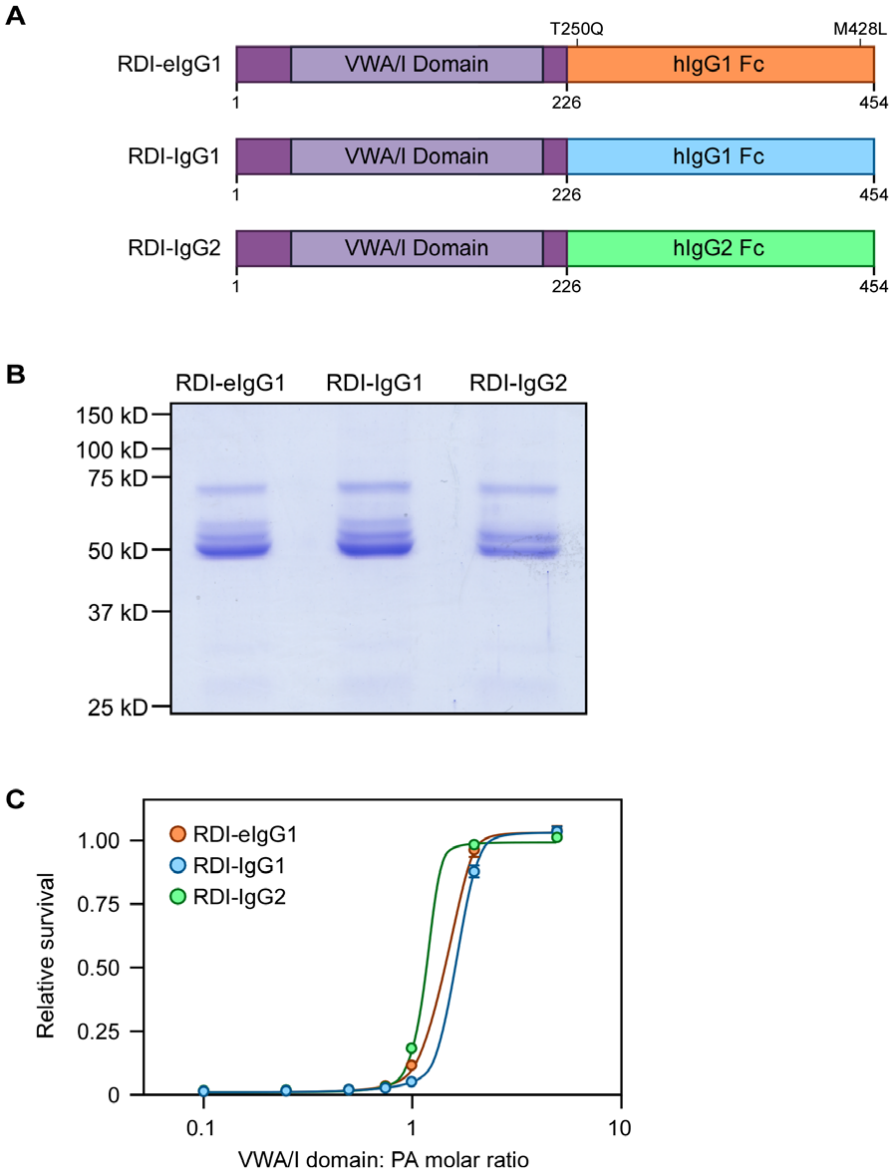
Construction of RDI fusion proteins with IgG1 or IgG2 Fc portions. A. Schematic of the RDI and Fc portions of the fusion proteins. B. SimplyBlue stained gel of RDI-eIgG1, RDI-IgG1, or RDI-IgG2 separated on 10% SDS-PAGE. Molecular weight markers are indicated. C. Inhibition of LeTx activity in RAW264.7 cells by RDI-eIgG1, RDI-IgG1, and RDI-IgG2.

Our RDI-IgG1 and RDI-IgG2 constructs (Fig. 6A) were engineered as described under Materials and Methods and expressed in the extracellular supernatants of transiently transfected 293 T cells. Both fusion proteins showed similar expression, molecular weights, and protein yields as the RDI-eIgG1 protein (Fig. 6B). The proteins also showed similar inhibitory concentrations compared to RDI-eIgG1 when tested in an *in vitro* intoxication assay on RAW264.7 cells (Fig. 6C).

**Figure 7:**
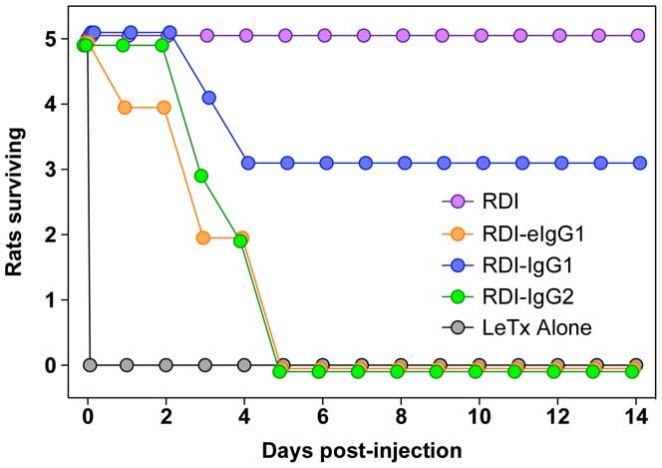
Delayed toxicity of rats following LeTx challenge with RDI-IgG1 and RDI-IgG2 fusion proteins. Male 200 gram HSD rats (5/group) were co-injected i.v. with LeTx (40 µg PA+12 µg LF) and either 19 µg of RDI; or 49 µg of either RDI-eIgG1, RDI-IgG1, or RDI-IgG2. Rats were monitored and time of death recorded.

The wild-type RDI-IgG1 RDI-IgG2 proteins were also tested *in vivo*, and again delayed toxicity was observed in each case (Figure 7). For each group, the animals survived for about 1 day, and by 4 days approximately half the animals in each group had died. As expected, co-administered RDI completely protected all animals (Fig. 7). These results demonstrate that the delayed toxicity that was initially seen with the RDI-eIgG1 protein is not due to the intrinsic extended circulation half-life of that protein relative to the other RDI-Ig-fusion proteins, but instead is a general feature of all the receptor decoy-IgG fusion proteins that were tested.

## Discussion

In this report we have tested the abilities of several different receptor decoy inhibitor-IgG fusion proteins to protect rats against challenge with a lethal dose of anthrax lethal toxin. The fusion proteins tested contained the Fc portions of IgG1, IgG2, or an altered form of IgG1 with increased circulation half-life. In each case, the inhibitor treatment significantly delayed, but did not prevent toxicity. Delayed toxicity was completely dependent upon the addition of LF since no toxicity was observed with the receptor decoy-Ig protein and PA alone. Delayed toxicity was associated with the assembly of long-lived LF/PA_63_ complexes on the receptor-Ig protein.

These data lead to a model in which the PA_83_ that was introduced into the rat bloodstream was processed by serum proteases [10], [11] and the resultant PA_63_ fragment assembled into oligomers on the receptor decoy-Ig protein where it bound LF. Alternatively, LF may bind to the PA_63_ oligomers prior to binding the RDI-Ig proteins. The LF/PA_63_ complexes were associated with long-lived RDI-IgG proteins, circulating in the bloodstream for an extended period of time. We speculate that, at a slow rate, the toxin complex dissociates from the long-lived inhibitor leading to death; the half-life of the PA-ANTXR2 VWA domain interaction is estimated to be ∼17 hours [19]. Although we found that protection is independent of Ig subclass (Figure 7), it is also possible that the toxin-receptor decoy-Ig proteins are taken up into cells by Fc receptor-mediated endocytosis and delivered to an acidic endosomal compartment where prepore-to-pore conversion is triggered and LF is introduced into the cellular cytoplasm. It may be possible to discriminate between these models using receptor-deficient mice, although in mice the kinetics of LeTx intoxication is significantly longer than in rats (e.g. Figure 2). Interestingly, regardless of the mode of internalization, the LF-PA complex would have to at least partially detach from the ANTXR2-portion to form a pore in the endosomal membrane [25].

Prior to this report there were two published studies using receptor decoy-Ig proteins as candidate anthrax therapeutics. The study of Viyusich *et al.* employed a receptor decoy-IgG2 protein comprised of the VWA domain of ANTXR2 fused to the Fc portion of human IgG2 [22]. In their report, the authors showed that the ANTXR2-IgG2 protein could protect DBA/2 mice against killing when administered 2 days after challenge with attenuated *B. anthracis* Sterne spores. In a separate study, Wycoff *et al.* employed a receptor decoy-IgG1 protein comprised of the VWA domain of ANTXR2 fused to the Fc portion of IgG1 [21]. They demonstrated that this protein could protect rabbits against death when it was administered up to 1 hour following an intranasal challenge with fully virulent *B. anthracis* Ames spores. Consistent with these findings, we have shown that treatment with the RDI-eIgG1 protein that was engineered to have an increased circulation half-life can protect A/J mice following intraperitoneal challenge with *B. anthracis* spores (Figure 2). The study of Viyusich and colleagues further showed that their inhibitor protected rats against anthrax lethal toxin challenge, at least 4 days following toxin exposure [22]. Animal survival at later time points following a fatal dose of anthrax lethal toxin was not reported in that study.

Taken together, these previous studies and the current report indicate that receptor decoy-Ig fusion proteins are attractive candidate anthrax therapeutics that can prevent death if administered shortly after bacterial infection: under these conditions, the decoy-Ig protein can presumably neutralize the immunosuppressive effects associated with low, sub-lethal toxin levels [26]. Receptor decoy-based inhibitors have the added benefit that they can neutralize wild-type, as well as altered, antibody-resistant, forms of the toxin [20], [21]. Therefore, these reagents may be particularly useful for early treatment of infection by vaccine-, antibiotic-, or antibody-resistant forms of *B. anthracis*. However, our data showing that these inhibitors did not provide long-term protection of rats challenged with a fatal dose of anthrax lethal toxin indicates that they may not be effective at preventing death at later time points of infection when fatal levels of toxin have already accumulated in the bloodstream. Studies are ongoing in our lab to overcome this limitation of these inhibitors by developing modified receptor decoys with intermediate circulation half-life properties since these altered forms of inhibitor might be expected to effectively block death at both early and late times following infection by *B. anthracis*.

## Materials and Methods

### Ethics Statement

Research was conducted in compliance with the Animal Welfare Act and other federal statutes and regulations relating to animals and experiments involving animals and adheres to the principles stated in the Guide for the Care and Use of Laboratory Animals, National Research Council, 1996. The facility where this research was conducted is fully accredited by the Association for Assessment and Accreditation of Laboratory Animal Care International. Animal research was conducted under approved protocol S09355 from the Institutional Animal Care and Use Committee, University of California, San Diego.

### Plasmid construction

The plasmid pRDI-eIgG1 was made by PCR-amplifying the ANTXR2 extracellular domain from pHS003 with the primers sCMG2For (5′-CTGCAGACCGGTGAGAGGATGGTGGCGGAG-3′) and sCMG2Rev (5′-ATCTAACCATGGTGGGCTGCAATTCTAGGAT-3′) to create a DNA fragment with an *Age*I site at the 5′ end and a *Nco*I site at the 3′ end. This fragment was ligated into pFUSE-hIgG1e1-Fc1 (Invivogen, San Diego CA) digested with *Age*I and *Nco*I to generate the expression plasmid for RDI-eIgG1. The sequence of the resultant plasmid was confirmed (Eton Bioscience Inc., San Diego CA). pRDI-IgG1 and pRDI-IgG2 were made by digesting pRDI-eIgG1 with Age1 and Nco1 to get the ANTXR2 insert and subcloning it between the *Age*I and *Nco*I sites of pFUSE-hIgG1-Fc1 or pFUSE-hIgG2-Fc1, respectively (Invivogen, San Diego CA). The sequences of both resultant plasmids were confirmed (Eton Bioscience Inc., San Diego CA).

### Protein expression and purification

The RDI-Ig proteins were produced by transient transfection in 293 T cells using the transfection reagent polyethylenimine (PEI, Polysciences Inc., Warrington PA; 25,000 MW, 1 mg/ml in H_2_0) in DMEM/1% FBS. 18 hours post-transfection, the extracellular media was changed and the supernatant containing the receptor decoy proteins was collected 48 hours later.

The proteins were purified from the crude supernatant using the Montage Antibody Purification Kit (Millipore # LSK2ABA20, Billerica MA). They were further concentrated using the Spin-X UF Concentrator (Corning # 431489, Lowell MA). Protein concentration was determined using the BCA Protein Assay Kit (Pierce # 23227, Rockford IL). The purified and concentrated proteins were analyzed by electrophoresis on a SDS-containing 10% polyacrylamide gel and visualized with SimplyBlue SafeStain (Invitrogen # LC6060, Carlsbad, CA). RDI was produced and purified from bacteria as previously described [18], [19].

### *In vitro* Intoxication Assay

Intoxication inhibition assays were performed in RAW264.7 cells as described previously [20]. RAW264.7 cells were originally obtained from the American Type Culture Collection (ATCC).

### Pharmacokinetics of RDI and RDI-Ig in rats

#### In vivo dosing

Male Harlan Sprague-Dawley (HSD) rats (180–200 g, Harlan, Indianapolis, IN) were anesthetized with isofluoranes and injected intravenously with either 900 µg of RDI or 50 µg of RDI-eIgG1 in PBS in a 500 µl final volume per rat. Blood was collected at various time points post-injection, up to 1 hour for rats dosed with RDI and up to 7 days for rats dosed with RDI-eIgG1. Blood samples were placed on ice upon collection and then centrifuged at 4°C at 12,000 RPM for 15 minutes. The plasma was collected and stored at −20°C until assayed.

#### ELISA quantitation and analysis

The half-lives of the RDI and RDI-eIgG1 proteins were determined by capture ELISA assay. Briefly, Immulon 2-HB microtiter plates (Dynex Technologies, Inc.) were coated with 1 µg per well of PA_83_ (List Labs), and then blocked with 3% non-fat dry milk in TBS (pH 7.0). Plasma samples taken from study animals and diluted 1∶10 in PBS were added to wells and incubated for 1 hour at room temperature. RDI and RDI-eIgG1 were mixed with 10% rat serum in PBS and used as standard controls. The standard and test samples were each assayed in triplicate. The plates containing the RDI samples were incubated at room temperature for 1 hour with a chicken anti-ANTXR2 antibody [27] diluted 1∶100 in 1% non-fat dry milk in TBS with 0.05% Tween 20, and then for 1 hour with biotin-conjugated goat anti-chicken antibody (Vector) diluted 1∶20,000 in 1% non-fat dry milk in TBS with 0.05% Tween 20. The plates containing the RDI-eIgG1 samples were incubated at room temperature for 1 hour with a biotin-conjugated goat anti-hIgG (Southern Biotech, Birmingham, AL) diluted 1∶20,000 in 1% non-fat dry milk in TBS with 0.05% Tween 20. All plates were incubated with streptavidin-alkaline phosphatase (GE Healthcare Bio-Sciences Corp., Piscataway, NJ) diluted 1∶5,000 in TBS for 45 minutes followed by a 20 minute incubation at 37°C with p-nitriphenyl phosphate substrate (Sigma-Aldrich, St. Louis, MO). Finally, 2 N NaOH was added and the absorbance values of each sample measured at 405 nm with a plate reader (Molecular Devices, Sunnyvale, CA). The average mean values of the triplicate samples were calculated and values of the standard sample without RDI or RDI-eIG1 was subtracted from each measurement. The measurements obtained with the known standard amounts of RDI and RDI-eIgG1 were graphed and the trendline equation was used to calculate the concentrations of the receptor decoy proteins in the different plasma samples as a function of time. Pharmacokinetic parameters were calculated using one-phase exponential decay (Graph Pad Prism, GraphPad Inc. La Jolla CA).

### In vivo intoxication assay in rats

Male 200 gram HSD rats (Harlan Laboratories, Indianapolis, IN) were co-injected i.v. with LeTx (40 µg PA+12 µg LF; established minimal lethal dose (MLD) [28]) and either PBS, 19 µg RDI, or 49 µg of either RDI-eIgG1, RDI-IgG1, or RDI-IgG2. These amounts of the receptor decoy proteins are equal to a 2∶1 molar ratio (VWA/I domain∶ PA). Rats were monitored and time of death recorded. Statistical analysis was performed using Prism (Graph Pad, Inc., La Jolla, CA)

### Toxicity testing RDI-eIgG1 in rats

Male 200 gram HSD rats (Harlan Laboratories, Indianapolis, IN) were injected i.v. with 123 µg RDI-eIgG1, which corresponds to a 5∶1 molar ratio of ANTXR2 VWI/I domain∶ PA. Blood samples were collected before injection and 15 hours and two days post injection and tested in a standard chemistry panel (Department of Animal Resources, The Scripps Research Institute.) Tissue was collected, sectioned, and stained with hematoxylin and eosin Y.

### Circulation and recovery of LeTx and RDI-eIgG1 *in vivo*

Male 200 gram HSD rats (Harlan Laboratories, Indianapolis, IN) were anesthetized with isofluoranes, pre-bled, and then injected through jugular vein cannulas with a total volume of 500 µl containing 40 µg PA, 12 µg LF, and 122 µg of RDI-eIgG1 in PBS. Blood was collected 15 hours after injection and serum was prepared and immediately used to precipitate RDI-eIgG1 using a protein G immunoprecipitation kit (Roche Diagnostics, Mannheim, Germany). The positive control used for this assay was serum from a non-injected control rat, spiked with 3.07 µg RDI-eIgG (Young Lab), 1.0 µg PA (List Biological Labs, Campbell, CA), and 0.3 µg LF(List Labs). The protein G agarose beads were incubated with 500 µl serum samples for 2 hours at room temperature with constant agitation, briefly spun down, and the beads were washed with 1 ml wash buffer four times. 50 µl Laemmli buffer containing ß-mercaptoethanol was added to each precipitated bead sample and the samples were vortexed, boiled for 10 minutes, briefly centrifuged, and the supernatants collected and stored at −20°C.

#### Virulence testing in the mouse infection model

Female A/J mice, approximately 8–10 weeks old, were challenged intraperitoneally (IP) *B. anthracis* Sterne strain spores, delivered in sterile water for injection. The mice received approximately 3.9×10^4^ spores and were then monitored for signs of clinical illness or death for 14 days after the challenge. These data were used to calculate the survival curves and mean times to death or euthanasia. The Sterne strain spores used for the challenge were prepared using Leighton and Doi medium as previously described [29].

#### Immunoblot for PA, RDI-eIgG1, and LF

Samples (100 ng) were analyzed by immunoblotting using NuPage 4–12% Bis-Tris 10 well mini gels with MOPS SDS running buffer (Invitrogen, Inc.). Positive control samples were prepared with 100 ng of either RDI-eIgG1, PA_83_, or LF contained in 15 µl loading buffer. A serum-only negative control sample was prepared by mixing equal parts rat serum from the pre-bleed samples with loading buffer. All samples were boiled for 5 minutes, loaded into wells of a 4–12% SDS-containing polyacrylamide gel and electrophoresed at 200 V for 45 minutes. The protein samples were transferred to PVDF membranes using a Trans-Blot cell (Bio-Rad) at 10 V overnight. Membranes were washed in PBS-Tween20 (PBST) and then blocked with 10% milk in PBST for 1 hour at room temperature. Primary antibodies were prepared in 10 ml 2% milk in PBST per membrane with either a 1∶1000 dilution of a chicken anti-ANTXR2 antibody [27] a 1∶250 dilution of a goat anti-PA antibody (List Laboratories, Campbell, CA), or a 1∶250 dilution of a goat anti-LF antibody (List Laboratories, Campbell, CA). Membranes were incubated with the primary antibody samples at 4°C overnight on a shaking platform, washed with PBST, and then incubated at room temperature for 1 hour with either HRP- anti-chicken, or HRP- anti-goat, secondary antibodies. Membranes were then washed with PBST and incubated with the SuperSignal West Pico chemiluminescent substrate (Thermo Scientific, Rockford, IL) for 5 minutes according to the manufacturer's instructions. Then membranes were then exposed to X-ray film for 5 mins at room temperature and developed.
